# Case Report: Mycobacterial epidural pyogranulomatous steatitis in a cat

**DOI:** 10.3389/fvets.2025.1610313

**Published:** 2025-06-04

**Authors:** Flora Decrop, Tiago Henriques, Annika Hermann, Caroline Fina, Patricia Álvarez, Juanjo Mínguez, Christoforos Posporis

**Affiliations:** ^1^Department of Neurology and Neurosurgery, Pride Veterinary Referrals, Independent Vetcare (IVC) Evidensia, Derby, United Kingdom; ^2^The Site Vet, Marlborough, United Kingdom; ^3^Department of Histology, Veterinary Pathology Group, Bristol, United Kingdom; ^4^Department of Diagnostic Imaging, Pride Veterinary Referrals, Independent Vetcare (IVC) Evidensia, Derby, United Kingdom

**Keywords:** feline, tetraparesis, myelopathy, infectious, extradural, spinal cord

## Abstract

A 2-year-old neutered male domestic short-haired cat was presented with chronic spinal hyperesthesia and a 24-h acute progression to non-ambulatory, non-painful tetraparesis, consistent with a C1-T2 myelopathy. Physical examination, serum biochemistry, hematology, thoracic and abdominal imaging were unremarkable. Magnetic resonance imaging revealed a well-defined, crescent-shaped, extra-dural, compressive, T2w/T1w/STIR hyperintense contrast-enhancing mass lesion within the dorsal and right lateral vertebral canal, from C2 to cranial C4. Mild mononuclear pleocytosis and increased protein concentration were detected on CSF analysis. Serology for Feline Corona Virus (FeCoV), *Toxoplasma gondii* IgM and IgG, *Cryptococcus* antigen, FIV/FeLV and CSF PCR for *T. gondii* and FeCoV were negative. A right-sided C2-C3 hemilaminectomy was performed, and the mass was debulked. Histopathology showed marked pyogranulomatous steatitis with multifocal lymphofollicular hyperplasia. Ziehl-Neelsen and PAS stains, as well as immunohistochemistry for FeCoV were negative. Positive amplicons with the DNA sequence most closely related to the genus *Mycobacterium* were detected on hsp65 gene-targeted PCR and sequencing of the resected tissue. The cat was treated with a one-week course of prednisolone, and was prescribed a six-month course of clarithromycin, pradofloxacin, and rifampicin, with clarithromycin being discontinued after 35 days due to poor patient compliance. A rapid and complete recovery was confirmed on re-examination at 2 weeks and no recurrence was reported at last follow-up, 20 months after diagnosis. This case represents one of the first documented instances of focal mycobacterial epidural steatitis in a cat, underscoring the importance of considering *Mycobacterium* infection in the differential diagnosis of epidural pathology. It also emphasizes the utility of PCR and subsequent sequencing for precise diagnosis. With appropriate treatment, a favorable long-term outcome is achievable.

## Introduction

Mycobacteria are acid-fast, rod-shaped, intracellular bacteria with a broad environmental distribution, capable of inducing a spectrum of manifestations in cats, including neurological disease. Based on their clinicopathological and biological characteristics, the most relevant mycobacteria in feline medicine are classified into three categories: the *Mycobacterium tuberculosis* complex (MTBC), non-tuberculous mycobacteria (NTM), and feline leprosy (1).

The MTBC group includes species such as *Mycobacterium tuberculosis* and *Mycobacterium bovis*, primarily associated with respiratory disease but also extrapulmonary infections, including cutaneous lesions. While domestic cats are generally resistant to *M. tuberculosis*, other MTBC members—particularly *Mycobacterium microti*—have emerged as significant pathogens implicated in clinical disease (2–5). Non-tuberculous mycobacteria, commonly found in the environment, act as opportunistic pathogens, causing cutaneous and systemic infections. Clinically relevant NTM species include those from the *Mycobacterium avium* complex (MAC) and other slow- and fast-growing species (1, 6). *Mycobacterium lepraemurium*, the causative agent of feline leprosy, is discussed separately due to its restriction to the skin (3).

Mycobacterial infections in cats manifest in cutaneous, visceral, ocular, skeletal, and neurological forms (2, 3, 7–12). Cutaneous lesions, the most common presentation, appear as nodular or non-healing wounds with draining tracts. Digestive forms may cause weight loss, vomiting, and diarrhea, while respiratory involvement may lead to various bronchial, interstitial, and alveolar infiltrates, hilar lymphadenopathy, pneumothorax, and pleural effusions resulting in dyspnea and coughing (2, 3, 7). Ocular disease is often part of disseminated mycobacteriosis, yet it may also present as a solitary manifestation with uveitis, blindness, and corneal, conjunctival and eyelid proliferative lesions (8, 9). Systemic dissemination may lead to fever, weight loss, ocular signs, hepatosplenomegaly, generalized lymphadenopathy, bone lesions, and central nervous system (CNS) involvement (2, 3, 7).

Neurological manifestations of mycobacterial infections vary depending on lesion localization. *Mycobacterium avium* and other species, including *M. bovis*, have been implicated in peripheral vestibular disease, intracranial infections, and pyogranulomatous meningoencephalitis (10–12), with vertebral osteomyelitis and paraspinal granulomas also reported (13, 14). However, to the best of our knowledge, focal myelopathy caused by an isolated epidural mycobacterial pyogranuloma has not been documented in cats. This case report describes the clinical presentation, imaging and histopathologic findings, treatment, and outcome of a domestic short-haired cat with focal mycobacterial epidural pyogranulomatous steatitis, highlighting the importance of considering this condition in cats with epidural pathology.

## Case description

A 2-year-old, male neutered, domestic, short-haired cat was presented with a 3-month history of spinal hyperesthesia, progressing over 24 h to non-ambulatory, non-painful tetraparesis. The cat had regular outdoor access, no history of travel, was housed alone, and was exclusively fed a dry commercial diet. Hematology performed 2 months prior showed mild lymphocytosis at 9.05 × 10^9/L (reference: 0.92–6.88 × 10^9/L), and serum biochemistry was normal. Thoracic radiographs did not reveal any abnormalities. The patient was treated with gabapentin (Summit, Summit Veterinary Pharmaceuticals Ltd., UK; 5 mg/kg PO q12h), meloxicam (Metacam, Boehringer Ingelheim, Germany; 0.05 mg/kg PO q24h), and exercise restriction for 4 weeks, resulting in significant improvement. No further treatment was administered during the subsequent 2 months, until the reported 24-h history of acute deterioration.

Physical examination was unremarkable. Neurological assessment revealed mild obtundation, non-ambulatory right-lateralized tetraparesis, and diminished postural reactions, more pronounced on the right side, particularly in the right thoracic limb. Withdrawal reflexes were reduced in both thoracic limbs, notably more in the right. All other spinal segmental reflexes, muscle mass and tone, and cranial nerve examination were normal. Spinal palpation elicited no discomfort. A right-sided C1-T2 myelopathy was suspected. Although mild obtundation was noted, it was believed to reflect nonspecific lethargy or discomfort; however, intracranial involvement could not be entirely excluded. The progressive course of clinical signs raised suspicion for infectious, inflammatory, or neoplastic etiologies, though a vascular complication (ischemic/hemorrhagic myelopathy) justifying the acute onset of severe neurologic deficits was also considered.

**Figure 1:**
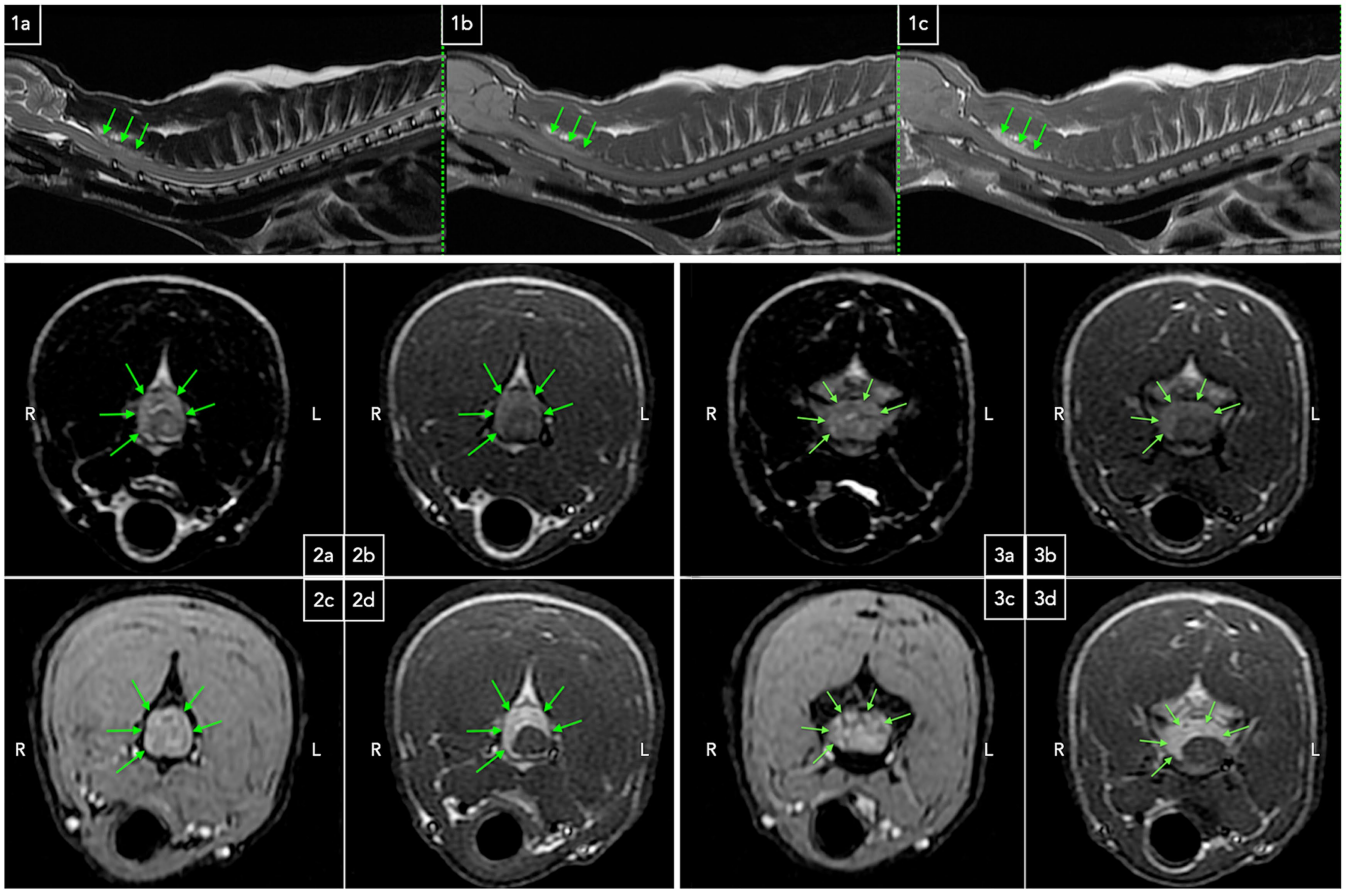
Sagittal and transverse MRI images of the cervicothoracic region demonstrating an extradural mass lesion. Sagittal T2w **(1a)**, T1w **(1b)**, and post-contrast T1w **(1c)** sequences depict an elongated extradural lesion that is heterogeneously hyperintense on T2w and T1w images compared to spinal cord parenchyma, with strong homogeneous contrast enhancement (green arrows). The lesion is dorsal to the spinal cord, effacing the dorsal cerebrospinal fluid and fat column, with suspected mild spinal cord compression. Transverse T2w **(2a)**, T1w **(2b)**, gradient echo **(2c)**, and post-contrast T1w **(2d)** sequences at the level of C2 vertebra reveal a crescent-shaped extradural T2w and T1w heterogeneously hyperintense strongly contrast enhancing lesion, predominantly occupying the right lateral and dorsal epidural space, with associated meningeal contrast enhancement (green arrows). The spinal cord appears T2w hyperintense **(2a)**. Transverse T2w **(3a)**, T1w **(3b)**, gradient echo **(3c)**, and post-contrast T1w **(3d)** sequences at the level of C3 vertebra demonstrate a similar extradural, moderately compressive lesion (green arrows). Punctate intralesional signal voids are visible on gradient echo images **(3c)**. Concurrent meningeal and dorsal vertebral arch contrast enhancement is noted, and the spinal cord exhibits T2w hyperintensity.

Repeated hematology and serum biochemistry profiles were unremarkable. Magnetic resonance imaging (MRI) was performed under general anesthesia using a 1.5 T scanner (SIGNA HDe, GE Healthcare). The protocol included sagittal T1-weighted (T1w), T2-weighted (T2w), and post-contrast T1w sequences, along with a sagittal T2w acquisition of the thoracolumbar vertebral column. Transverse sequences consisted of T1w, T2w, gradient echo, and post-contrast T1w images. A dorsal cervicothoracic short tau inversion recovery (STIR) sequence was also obtained. Post-contrast imaging was conducted using gadoteric acid (Dotarem®, Guerbet, France; 0.1 mmol/kg IV). A well-defined, crescent-shaped, extradural mass lesion located within the dorsal and right lateral vertebral canal, extending from C2 to cranial C4 vertebrae, and causing moderate spinal cord compression, was identified. Compared to normal spinal cord parenchyma, the lesion was heterogeneously hyperintense on T2w, T1w, and STIR sequences, exhibited homogeneous, strong contrast enhancement, and contained intralesional punctate areas of signal void. Associated meningeal enhancement was observed, along with increased contrast uptake in the vertebral arch of C3 (Figure 1). No abnormalities suggestive of systemic disease were identified. Neoplastic etiologies (e.g., lymphoma) were suspected based on imaging characteristics, while infectious causes (e.g., protozoal, fungal, bacterial) were regarded as less probable.

Lumbar cerebrospinal fluid (CSF) analysis showed a total nucleated cell count of 8/μl (reference: 0–5/μl), 4,160 red blood cells/μl, and elevated protein at 110.5 mg/dL (reference: <45 mg/dL). Cytology indicated mild mononuclear pleocytosis despite suspected iatrogenic hemodilution. Polymerase chain reaction (PCR) for *Toxoplasma gondii* and feline coronavirus (FeCoV) in CSF, *Cryptococcus neoformans* antigen, serology for *T. gondii* and FeCoV, and FeLV/FIV SNAP test were negative. Abdominal ultrasound showed no evidence of disseminated disease.

Given the absence of multicentric disease, a right-sided C2-C3 hemilaminectomy was performed via a lateral approach for sampling and decompressive purposes (15). A friable, poorly demarcated, pale tan to yellow, infiltrative, extradural mass lesion involving the epidural fat was identified and debulked, resulting in visible decompression of the spinal cord. Samples were submitted for histopathology, but bacteriological cultures were not requested. The cat remained hospitalized for 2 days, and received methadone (Comfortan, Dechra Veterinary Products Ltd., UK; 0.2 mg/kg IV q4h), cefuroxime (Zinacef, Sandoz Ltd., Switzerland; 20 mg/kg IV q8h), and dexamethasone (Dexadreson, MSD Animal Health Ltd., UK; 0.1 mg/kg IV q24h).

At discharge, the cat was ambulatory tetraparetic with proprioceptive ataxia in all limbs. Gabapentin (Summit, Summit Veterinary Pharmaceuticals Ltd., UK; 10 mg/kg PO q8h), cephalexin (Therios, Ceva Animal Health Ltd., France; 20 mg/kg PO q12h), and prednisolone (Prednidale, Dechra Veterinary Products Ltd., UK; 0.5 mg/kg PO q24h) were prescribed and the owner was advised to restrict high-impact activity. Two weeks later, the neurological examination had normalized.

**Figure 2:**
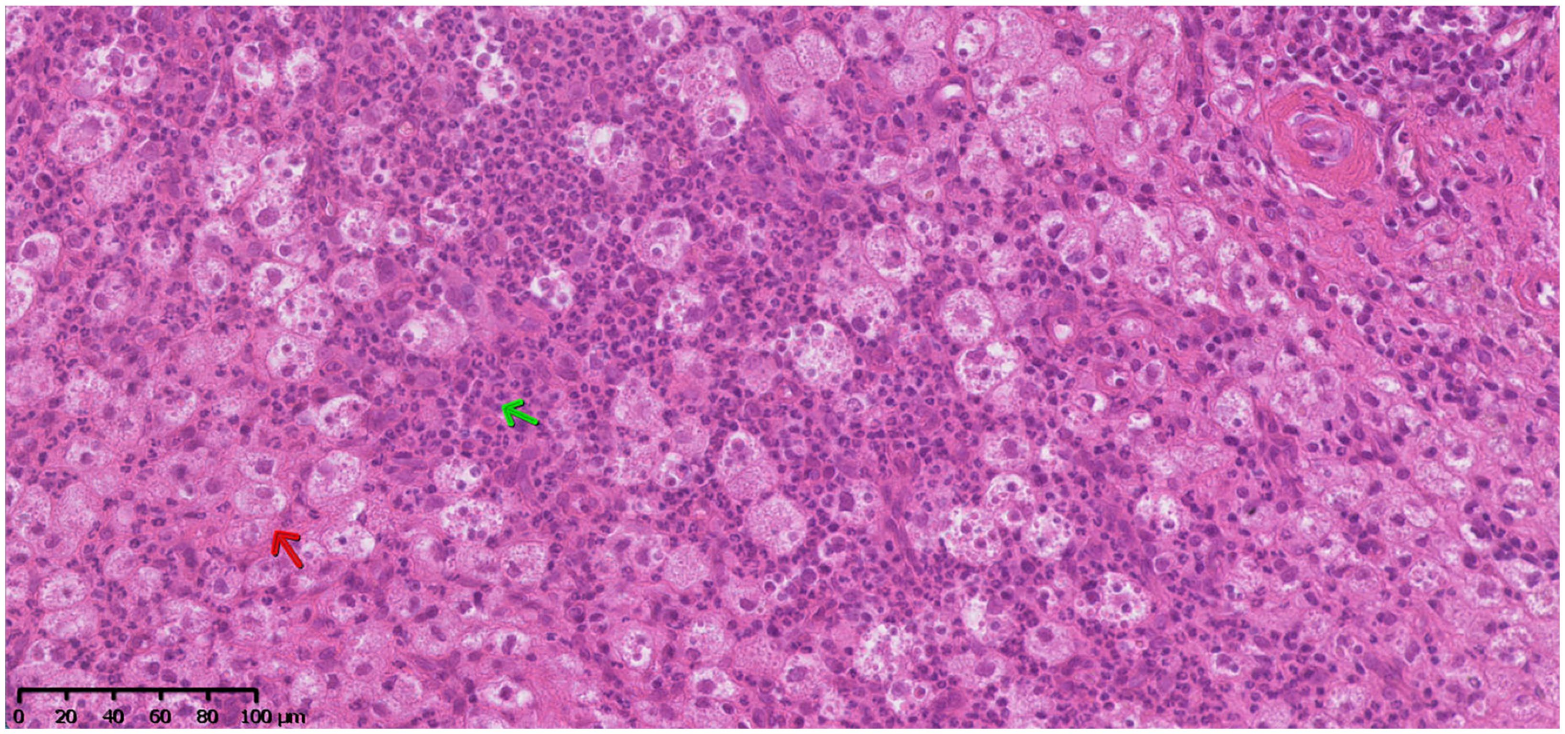
Hematoxylin and eosin (H&E) stain. Microscopic image of the resected lesion involving the epidural fat at C2-C3 showing marked pyogranulomatous steatitis (green arrow = neutrophils, red arrow = macrophages).

Histopathological examination of the resected epidural tissue revealed extensive pyogranulomatous inflammation (Figure 2). Findings included diffuse steatitis, multifocal moderate lymphofollicular hyperplasia, and peripheral reactive lymphoid follicles without atypical cells. Reactive bony remodeling was noted, with normal bone marrow organization. Immunohistochemistry for FeCoV was negative. Periodic acid-Schiff (PAS) and Ziehl-Neelsen stains did not reveal mycobacteria or fungal organisms. A DNA sequence most closely related to the genus *Mycobacterium* (Query cover 90%, identity 98%) was identified by hsp65 gene-targeted PCR and subsequent basic local alignment search tool (BLAST) analysis against sequences in the GenBank database.

Mycobacterial epidural steatitis was diagnosed 20 days after presentation, and treatment was initiated after informing the owner of the zoonotic risk. A multi-drug antibiotic regimen was initiated comprising rifampicin (Rifadin, Sanofi UK Ltd., UK; 10 mg/kg PO q24h), pradofloxacin (Veraflox, Bayer Animal Health Ltd., Germany; 3.5 mg/kg PO q24h), and clarithromycin (Klaricid, AbbVie Ltd., UK; 10 mg/kg PO q12h), following published therapeutic guidelines for feline mycobacteriosis (2). Clarithromycin was discontinued after 35 days due to poor patient compliance. Pradofloxacin and rifampicin were continued for 6 months (2).

One month after presentation, the neurological examination was normal, though pruritus, crusted skin lesions, and mild alopecia on the dorsal neck were noted. A dermatological referral was declined, and topical chlorhexidine-based antibacterial treatment was initiated. By day 81 of antibiotic therapy, there were no neurological or respiratory signs but a cystic cutaneous lesion on the chin was identified. Thirteen months post-diagnosis, the cat developed congestive heart failure due to hypertrophic cardiomyopathy and was treated successfully with no recurrence of cardiac, cutaneous, or neurological signs during the last 7 months of follow-up.

## Discussion

This case report describes the clinicopathologic and imaging findings of a young cat from the United Kingdom (UK) diagnosed with mycobacterial epidural pyogranulomatous steatitis. The incidence of mycobacterial infections in cats is estimated at 1%, but clinically significant infections are increasingly more common in the UK (16). In the present case, molecular diagnostics identified *Mycobacterium* spp., underscoring the importance of considering this pathogen in cases of unexplained pyogranulomatous inflammation, particularly in cats with outdoor access, raw diets, or other risk factors (16, 17).

Focal epidural pyogranulomatous steatitis secondary to mycobacterial infection has not been previously documented in cats, contrasting documented cases of spinal mycobacteriosis with demonstrated systemic dissemination (13, 14). These include *Mycobacterium kansasii* infection causing sacral and coccygeal osteolysis (13), and *M. microti* presenting as an intradural extramedullary granuloma with concurrent subcutaneous nodules, pulmonary infiltrates, and pyogranulomatous splenitis (14). Other prior reports include pyogranulomatous meningoencephalitis caused by *Mycobacterium avium* subsp. *Hominissuis* (12), and a focal intracranial lesion caused by *M. avium* (11). Additionally, *M. avium* has been implicated in granulomatous inflammation at the left ear base, associated with vestibular dysfunction and ipsilateral facial paralysis (10), as well as in mycobacterial neuritis (18). In our patient, multifocal CNS disease was neither clinically suspected nor identified on diagnostic imaging; however, chronic, non-specific spinal hyperesthesia preceded the acute onset of tetraparesis, and mild obtundation was suspected at presentation, raising the possibility of undetected dissemination.

Cutaneous forms are most frequently observed in feline mycobacterial infections (2, 3, 6, 7). Initially, our patient was diagnosed with focal disease, with no additional pathology detected on physical examination and thoracic radiographs or abdominal ultrasonography. Subsequently, the cat developed cutaneous lesions of unknown origin, raising the possibility of either dissemination to the skin or an adverse reaction to rifampicin, both of which have been previously reported (19). In this case, topical antibacterial therapy was initiated without additional diagnostic investigations or discontinuation of rifampicin, leaving both theories unproven. Reported species causing cutaneous lesions include *Mycobacterium fortuitum*, *M. bovis*, *M. microti*, *M. lepraemurium,* and *M. avium* (3, 6, 7), with *M. microti* (14) and *M. avium* (10–12) also associated with neurological involvement. While it is possible that one of these species was involved in our case, this remains speculative due to the absence of dermatological and additional molecular investigations. The route of infection is also unclear. In cats, mycobacteriosis can result from wound contamination, gastrointestinal or respiratory transmission, or contact with rodents (3, 6, 7). Known risk factors include hunting behavior (3, 20–22), exposure to infected cattle (6, 11), and raw meat diets (17), none of which were identified in this patient.

*Mycobacterium tuberculosis* is the primary cause of vertebral granulomatous infections in humans (23). Spinal epidural abscesses are commonly associated with predisposing conditions such as diabetes mellitus, chronic renal failure, cancer, or immunodeficiency (24), none of which were identified in the present case. Localized back pain is the most common symptom, while neurological deficits are rarely the initial presentation (24, 25). Similarly, our patient suffered from spinal hyperesthesia alone for 3 months, but ultimately the signs progressed to neurological deficits. A previously reported case of feline disseminated mycobacteriosis with spinal involvement also presented with signs of discomfort and neurological dysfunction, including over-grooming of the lumbar area and distal tail, increased tail swishing, and proprioceptive ataxia in the pelvic limbs (14).

In humans, MRI is the preferred modality for diagnosing spinal tuberculosis, providing detailed visualization of soft tissue involvement, abscess extension, and spinal cord compression (24–26). Gadolinium-enhanced MRI aids in distinguishing tuberculosis from other causes of infective spondylodiscitis (25), with meningeal contrast uptake and rim enhancement of osseous and paraspinal abscesses being uncommon in nontuberculous cases (26). Typical findings include intervertebral disc involvement, early vertebral bone marrow T1w hypointense and STIR hyperintense lesions suggestive of edema, post-contrast vertebral enhancement, and paravertebral soft tissue changes. Epidural expansion occurs in 62.5% of cases (27), leading to spinal cord compression, with lesions appearing T1w hypointense and T2w/STIR hyperintense (27, 28). While our case shared some of these imaging characteristics, notable differences included the cervical rather than the typical lumbar location observed in humans (27, 28), absence of intervertebral disc and paravertebral soft tissue involvement, and distinct T1w hyperintensity of the epidural lesion, likely reflecting pathological fat content. Punctate signal voids within the lesion, initially considered hemorrhagic foci, were not confirmed histopathologically and may represent vascular structures. Comparable MRI characteristics were reported in a feline disseminated mycobacteriosis case with a lumbar intradural-extramedullary lesion (14), and the absence of surgical confirmation leaves extradural localization a possible but unverified consideration.

The diagnosis of spinal mycobacteriosis was established through hsp65 gene-targeted PCR and sequencing of the epidural mass, pursued after histopathological examination showing pyogranulomatous inflammation. This analysis revealed positive amplicons with DNA sequences closely related to the genus *Mycobacterium*. The hsp65 gene is a validated target for PCR-based identification of mycobacteria. When combined with sequencing and comparison to reference databases, this method achieves high specificity, typically between 98 and 100%, with a false-positive rate of less than 1% (29). Both PAS and Ziehl-Neelsen stains were negative, but this could suggest a paucibacillary infection, where the bacterial load is too low for reliable identification via standard staining techniques (30). Gunn-Moore et al. (16) reported that although 1% of feline tissue samples from the UK exhibited histopathological changes suggestive of mycobacterial infection, only 0.3% were confirmed as Ziehl-Neelsen positive, underscoring the limited sensitivity of this method in detecting mycobacteria.

The specific *Mycobacterium* species involved was not determined. While culturing fresh tissue samples could have aided in species identification, many mycobacterial species are slow-growing or may not grow at all. Nevertheless, bacteriological cultures should have been performed, and this represents a diagnostic limitation in the present case. The interferon-gamma release assay (IGRA) is another useful diagnostic tool that evaluates the cell-mediated immune response to mycobacterial infections with only 2 mL of heparinized blood (31). Although IGRA shows good sensitivity for infections caused by MBTC and can differentiate between *M. bovis* and *M. microti*, it has limitations in distinguishing between various other mycobacterial species (31). Given these constraints, molecular techniques such as PCR sequencing remain essential for diagnosing mycobacterial infections, especially in paucibacillary cases where traditional methods may fail (16, 30). Although species identification was not achieved in this case, the combination of histopathology and PCR provided compelling evidence for spinal mycobacteriosis, highlighting the need for advanced diagnostic approaches in similar cases.

A differential diagnosis for the histopathological findings in the present case was epidural idiopathic sterile pyogranulomatous inflammation. Although this condition has been documented in dogs, particularly Dachshunds (32–34), and in a single feline case (35), prior reports did not include comprehensive investigations for mycobacterial organisms, as PCR testing was not performed on the resected pyogranulomas. Moreover, most affected dogs experienced recurrent episodes, raising concerns about systemic dissemination and persistent disease despite initial surgical improvement (32, 33). In contrast, surgical intervention resulted in good recovery in the feline case, but the follow-up period was relatively short at 3 months (35). Consequently, the possibility of an undetected mycobacterial etiology cannot be entirely excluded. These findings underscore the importance of a multimodal diagnostic approach, integrating molecular, histopathological, and immunological techniques, to enhance the detection and characterization of mycobacterial infections in veterinary patients.

Currently, no drugs are officially approved for the treatment of feline mycobacteriosis (7, 36). It is generally recommended to use a combination of two or three antibiotics over several months to improve treatment efficacy and reduce the risk of antimicrobial resistance (2, 3, 7). In human medicine, surgical intervention for spinal tuberculosis is indicated when neurological deficits are present, spinal cord compression exceeds 50%, epidural abscesses are observed, severe pain is reported, medical treatment fails to control the disease, and/or tissue diagnosis is necessary (23, 24). Our feline patient exhibited significant neurological deficits and spinal cord compression, justifying surgical decompression and, crucially, enabling a definitive diagnosis. Surgical management in human patients provides benefits such as improved early neurological recovery, and reduced recurrence rates (25). While our patient showed rapid neurological improvement, the extent to which this was influenced by surgical decompression remains uncertain.

Our patient developed congestive heart failure secondary to hypertrophic cardiomyopathy 7 months after treatment interruption. The association between clarithromycin and cardiovascular events has been investigated in human medicine (37–39). One study reported an increased risk of myocardial infarction and arrhythmia during *Helicobacter pylori* eradication therapy containing clarithromycin, though no elevated risk was observed after treatment cessation (37). This makes it unlikely that clarithromycin contributed to the development of congestive heart failure in our case, as it occurred a year after discontinuation. Furthermore, You et al. found no significant link between clarithromycin use and long-term all-cause mortality, regardless of cardiovascular comorbidities (38). Similarly, Alispahic et al. found no increased risk of major adverse cardiovascular events, all-cause mortality, or cardiovascular death in patients treated with clarithromycin or other macrolides compared to amoxicillin over 3 years (39).

The prognosis for feline mycobacteriosis depends on the mycobacterial species, disease severity, and timing of treatment (2, 3, 7, 40). Disseminated infections have a worse outcome, requiring prolonged multidrug therapy and carrying a risk of relapse (3, 40), whereas localized cutaneous forms respond well to early intervention (2, 6, 36). Reported cases of intracranial infection were linked to poor outcomes, while peripheral vestibular disease and spinal mycobacteriosis were associated with good long-term recovery (10–14). In our case, the cat remained neurologically normal 20 months post-diagnosis, further supporting the potential for favorable outcomes in spinal mycobacterial infections. Nevertheless, due to the zoonotic potential of certain species, careful management is essential, especially for immunocompromised individuals or those at risk of aerosol transmission (41).

## Conclusion

Mycobacterial infection should be considered a differential diagnosis in cases of pyogranulomatous epidural pathology, even in the absence of systemic dissemination. Notably, Ziehl-Neelsen and PAS staining may yield false-negative results; therefore, PCR testing, in conjunction with other diagnostic modalities, is essential for accurate identification. This is particularly relevant given the potential for favorable outcomes with appropriate antimicrobial therapy. In our case, rapid neurological improvement followed surgical debulking and spinal cord decompression. However, further research is needed to elucidate the relationship between early surgical intervention and the rate of neurological recovery.

## Data Availability

The original contributions presented in the study are included in the article/supplementary material, further inquiries can be directed to the corresponding author.
